# Long-term depression is differentially expressed in distinct lamina of hippocampal CA1 dendrites

**DOI:** 10.3389/fncel.2015.00023

**Published:** 2015-02-05

**Authors:** Binu Ramachandran, Saheeb Ahmed, Camin Dean

**Affiliations:** Trans-synaptic Signaling Group, European Neuroscience InstituteGoettingen, Germany

**Keywords:** hippocampus, long-term depression, learning, memory, synaptic plasticity

## Abstract

Information storage in CA1 hippocampal pyramidal neurons is compartmentalized in proximal vs. distal apical dendrites, cell bodies, and basal dendrites. This compartmentalization is thought to be essential for synaptic integration. Differences in the expression of long-term potentiation (LTP) in each of these compartments have been described, but less is known regarding potential differences in long-term depression (LTD). Here, to directly compare LTD expression in each compartment and to bypass possible differences in input-specificity and stimulation of presynaptic inputs, we used global application of NMDA to induce LTD. We then examined LTD expression in each dendritic sub-region—proximal and distal apical, and basal dendrites—and in cell bodies. Interestingly, we found that distal apical dendrites exhibited the greatest magnitude of LTD of all areas tested and this LTD was maintained, whereas LTD in proximal apical dendrites was not maintained. In basal dendrites, LTD was also maintained, but the magnitude of LTD was less than in distal apical dendrites. Blockade of inhibition blocked LTD maintenance in both distal apical and basal dendrites. Population spikes recorded from the cell body layer correlated with apical dendrite field EPSP (fEPSP), where LTD was maintained in distal dendrites and decayed in proximal dendrites. On the other hand, LTD of basal dendrite fEPSPs was maintained but population spike responses were not. Thus E-S coupling was distinct in basal and apical dendrites. Our data demonstrate cell autonomous differential information processing in somas and dendritic sub-regions of CA1 pyramidal neurons in the hippocampus, where LTD expression is intrinsic to distinct dendritic regions, and does not depend on the nature of stimulation and input specificity.

## Introduction

Long-term potentiation (LTP) and long-term depression (LTD) of synaptic strength are thought to represent the cellular mechanism by which learning and memory formation occur (Bliss and Collingridge, 1993; Malenka, 1994b). The mechanisms underlying LTD are not as well understood as those of LTP, but both processes promote the storage of memories (Heynen et al., 1996; André and Manahan-Vaughan, 2013). The spatial pattern and amount of Ca^2+^ entering synaptic sites determines the nature of plasticity in terms of either LTP or LTD (Cummings et al., 1996; Hansel et al., 1996, 1997). NMDA receptors are essential for learning and memory (Morris et al., 1986; McHugh et al., 1996; Tsien et al., 1996). In the hippocampus NMDA receptors are crucial for LTP and LTD at the Schaffer collateral-pyramidal synapse of the CA1 region—one of the best-studied plasticity models in the mammalian brain (Dudek and Bear, 1992; Mulkey and Malenka, 1992).

Hippocampal CA1 pyramidal neurons have distinct compartmentalized domains with distinct inputs, signaling cascades and plasticity mechanisms thought to be crucial for synaptic integration (Spruston, 2008). These domains consist of basal dendrites extending into the stratum oriens, the pyramidal cell body layer in stratum pyramidale, proximal apical dendrites and distal apical dendrites in stratum radiatum. LTP is differentially expressed in distinct dendritic laminae. For example, the threshold for LTP is lower in proximal apical dendrites than in distal apical dendrites (Sajikumar and Korte, 2011), while the threshold for LTP in basal dendrites is reportedly similar to that in distal apical dendrites (Sajikumar et al., 2007), although different stimulation protocols were used in each region in the latter report. In addition, in a comparison of dopamine-induced LTP in basal and distal apical dendrites, expression of LTP in basal dendrites required L-type voltage-gated calcium channels, and expression of LTP in distal apical dendrites required BDNF (Navakkode et al., 2012).

Less is known about the lamina specificity of LTD. LTD was maintained in distal apical dendrites, but not in proximal dendrites in one study (Parvez et al., 2010), and in another no difference was found between apical and basal dendrite LTD, but proximal and distal dendrites were not distinguished (Pavlowsky and Alarcon, 2012). Differences in LTD expression in distinct dendritic branches (apical vs. basal) or lamina (stratum radiatum vs. stratum oriens) of CA1 neurons independent of input specificity, has not been explored.

LTD can be induced in the hippocampus by chemical or low frequency electrical stimulation (Malenka, 1994a; Bear and Abraham, 1996; Lee et al., 1998; Kamal et al., 1999; van Dam et al., 2002). Chemical LTD induced by application of 20–50 μM NMDA for 3–5 min occludes further induction of electrical LTD (Oliet et al., 1997; Kameyama et al., 1998; Lee et al., 1998; Kamal et al., 1999; Li et al., 2004) and is therefore thought to be mechanistically identical to electrically induced LTD. To investigate potential differences in the expression of LTD in distinct regions of CA1 pyramidal neuron dendrites and cell bodies we induced LTD with 30 μM NMDA for 5 min. NMDA-induced LTD has the advantage of activating all synapses in all sub-regions of the hippocampus identically and therefore bypasses potential differences in inputs to each region. This further allows distinction of post-synaptic cell-intrinsic differences in LTD expression in distinct dendritic branches of CA1 neurons.

## Materials and methods

All research involving animals was approved by and done in accordance with the Institutional Animal Care and Ethics Committees of Goettingen University (T10.31), and in accordance with German animal welfare laws. All efforts were made to minimize the number of animals used and their suffering.

## Hippocampal slice preparation

Slice preparation and recovery were carried out as described previously (Ramachandran and Frey, 2009). Eight-week-old male C57BL/6 mice purchased from Charles River were anesthetized with isoflurane and decapitated. The hippocampus was removed from the brain and 400 μM thick slices were cut transversely from the dorsal hippocampus in ice-cold artificial cerebrospinal fluid (ACSF) containing (in mM) 124 NaCl, 4.9 KCl, 1.2 KH_2_PO_4_, 2.0 MgSO_4_, 2.0 CaCl_2_, 24.6 NaHCO_3_, 10.0 D-glucose (saturated with 95% O_2_ and 5% CO_2_, pH 7.4, 305 mOsm), using a tissue chopper (Stoelting).

## Electrophysiology

**Figure 1:**
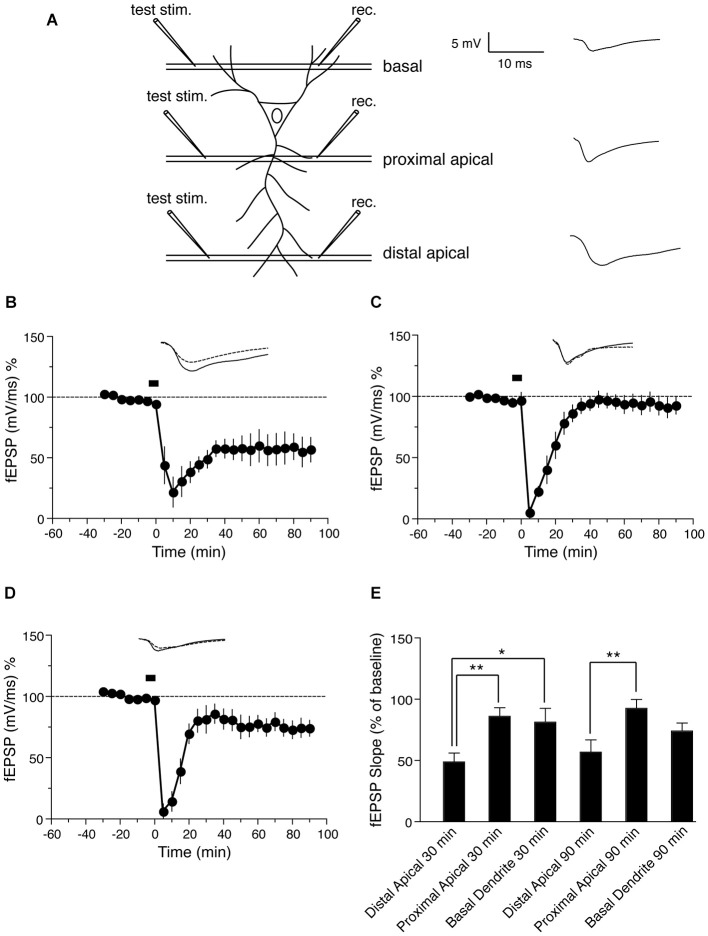
**NMDA-induced LTD in distinct dendritic sub-regions of hippocampal CA1 neurons. (A)** Schematic representation of transverse hippocampal CA1 lamina with test pulse stimulation electrode locations in proximal, distal and basal dendritic regions, and the recording electrode location for recording fEPSPs from the corresponding regions. The analog trace is a representative example of recorded potentials, and the scale is the same for all following panels. **(B)** Time course of the change in the slope of the fEPSP recorded from distal apical dendrites after induction of LTD by application of 30 μM NMDA for 5 min. Drug application is indicated with a black bar (*n* = 6). **(C)** Time course of the change in slope of the fEPSP recorded from proximal apical dendrites after NMDA-induced LTD. Black bar indicates the time NMDA was applied (*n* = 6). **(D)** Time course of the change in fEPSP slope recorded from basal dendrites after NMDA-induced LTD. Black bar indicates the time NMDA was applied (*n* = 7). **(E)** Comparison of NMDA induced LTD in distal, proximal and basal dendrites. The change in fEPSP is expressed as the percentage change from baseline. Analog traces represent typical fEPSPs from 30 min before (solid line) and 90 min after (dashed line) NMDA application. Scale bar for all analog traces is 5 mV/10 ms as shown in panel **(A)**.

Slices were incubated in an interface chamber (volume 2 ml) at 32°C and high oxygen tension was maintained by bubbling 95% O_2_ and 5% CO_2_ (30 l/h). Slices were perfused at 0.7 ml/min with ACSF. Slices were allowed to recover for 3 h after preparation. Then monopolar lacquer-coated stainless steel electrodes (571000, A-M Systems), used for recording and stimulating, were positioned in the CA1 region, as shown in Figures 1A or 4A, for different experiments. The population spike amplitude (PSA) and field EPSP (fEPSP) slope were recorded with a Model 1700 differential AC amplifier (A-M Systems) and Power 1401 analog-to-digital converter (Cambridge Electronic Design), and monitored on-line with the custom-made software, PWIN (IFN, Magdeburg). After the incubation period, the test stimulation strength was determined for each input to elicit 25% of the PSA or 40% of the fEPSP slope function for LTD induction. Baseline recording began at least 3.30 h after preparation, using test stimuli consisting of four biphasic constant current pulses (*f* = 0.2 Hz; pulse duration: 0.1 ms/polarity; averaged on-line) per time point, every 5 min for a minimum period of 30 min. 30 μM NMDA was bath applied for 5 min to induce LTD. Electrical LTD was induced with a low-frequency stimulus protocol of 900 bursts, where each burst consisted of three stimuli at 20 Hz, with an interburst interval of 1 s and a stimulus duration of 0.2 ms per half-wave, for a total of 2700 stimuli at 1 Hz. After drug application or low frequency stimulation (LFS), test stimuli were delivered every 5 min for up to 90 min.

**Figure 2:**
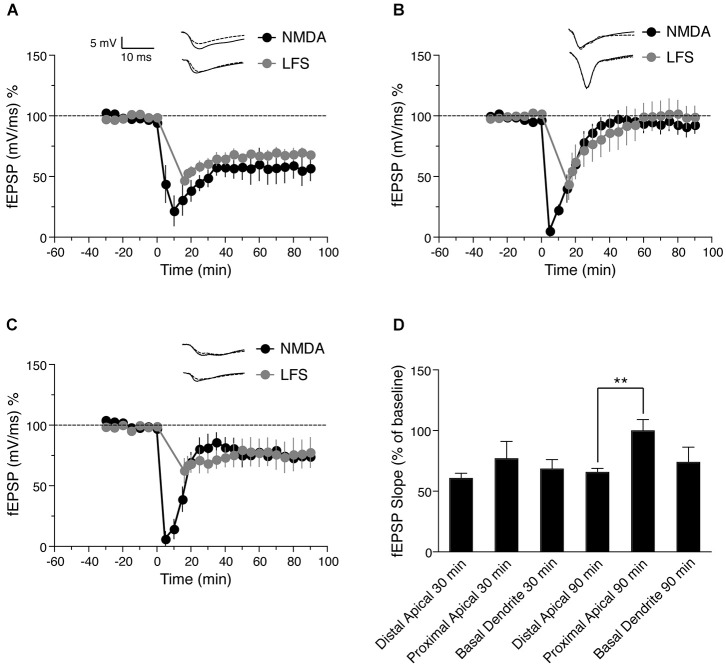
**Comparison of NMDA and low frequency stimulation (LFS)—induced LTD in distinct dendritic sub-regions of hippocampal CA1 neurons. (A)** Time course of the change in the slope of the fEPSP recorded from distal apical dendrites after induction of LTD by application of 30 μM NMDA for 5 min (black circles, *n* = 6) or after induction of LTD by LFS (gray circles, *n* = 5). **(B)** Time course of the change in slope of the fEPSP recorded from proximal apical dendrites after NMDA-induced LTD (black circles, *n* = 6) or LFS-induced LTD (gray circles, *n* = 6). **(C)** Time course of the change in fEPSP slope recorded from basal dendrites after NMDA-induced LTD (black circles, *n* = 7) or LFS-induced LTD (gray circles, *n* = 4). **(D)** Comparison of LFS-induced LTD in distal, proximal and basal dendrites. The change in fEPSP is expressed as the percentage change from baseline. Analog traces represent typical fEPSPs from 30 min before (solid line) and 90 min after (dashed line) NMDA application or and LFS. Scale bar for all analog traces is 5 mV/10 ms as shown in panel **(A)**.

**Figure 3:**
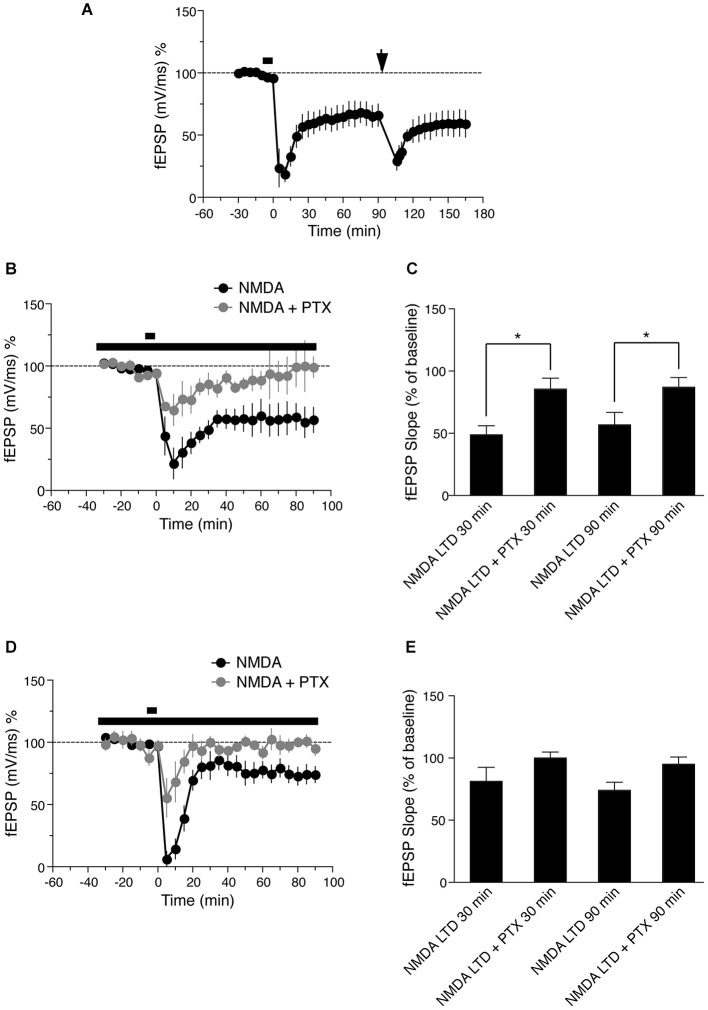
**(A)** Time course of the change in the slope of the fEPSP recorded from distal apical dendrites after induction of LTD by application of 30 μM NMDA for 5 min. Drug application is indicated with a black bar. Ninety minutes after LFS LTD was induced (LFS, indicated by black arrow, *n* = 5). **(B)** Time course of the change in slope of the fEPSP recorded from distal apical dendrites in presence of NMDA (black circles, *n* = 6) or 50 uM picrotoxin (PTX) and NMDA gray circles (*n* = 4). The long lack bar indicates the time PTX was applied and the short bar indicates the time NMDA was applied. **(C)** Comparison of NMDA induced LTD in presence of PTX in distal dendrites. The change in fEPSP is expressed as the percentage change from baseline. **(D)** Time course of the change in slope of the fEPSP recorded from basal dendrites in presence of NMDA (black circles, *n* = 7) or 50 uM PTX and NMDA (gray circles, *n* = 4). The long lack bar indicates the time PTX was applied and the short bar indicates the time NMDA was applied. **(E)** Comparison of NMDA induced LTD in presence of PTX in basal dendrites. The change in fEPSP is expressed as the percentage change from baseline.

**Figure 4:**
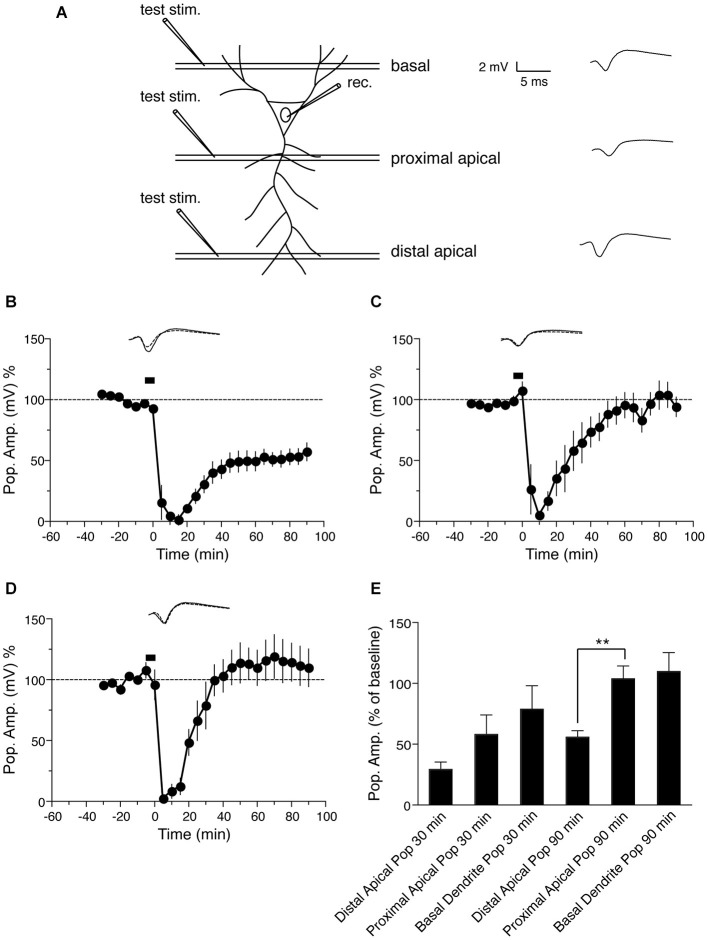
**NMDA-induced LTD in the the population spike response in CA1 neurons. (A)** Schematic representation of transverse hippocampal CA1 lamina with test pulse stimulation electrode locations in proximal and distal dendrites of the stratum radiatum and in basal dendrites of the stratum oriens, and the location of the recording electrode for the population spike from the cell body layer of CA1 pyramidal neurons. The analog trace is a representative example of recorded potentials, and the scale is the same for all following panels. **(B)** Time course of change in the amplitude of the population spike recorded from the cell body layer, when the test pulse stimulation electrode was placed in the distal apical dendrite sub-region, following induction of LTD by application of 30 μM NMDA for 5 min. Drug application is indicated with a black bar (*n* = 6). **(C)** Time course of the change in the population spike response recorded from the cell body layer when the test pulse stimulation electrode was placed in the proximal apical dendrite region in stratum radiatum during NMDA-induced LTD. Black bar indicates the time NMDA was applied (*n* = 6). **(D)** Time course of the change in the population spike response recorded from the cell body layer when the test pulse stimulation electrode was placed in stratum oriens during NMDA-induced LTD. Black bar indicates the time NMDA was applied (*n* = 7). **(E)** Comparison of NMDA-induced population spike response recorded from the cell body layer, with test pulse stimulation electrodes placed in the indicated regions of the CA1. The change in the population spike is expressed as the percentage change from baseline. Analog traces represent typical population spikes 30 min before and 90 min after NMDA application. Scale bar for all analog traces is 2 mV/5 ms as shown in panel **(A)**.

## Pharmacological substances

NMDA was purchased from Merck Pharma, Germany and all other chemicals were purchased from Sigma.

## Statistical analysis of electrophysiological data

Average values of the slope of the fEPSP per time point (expressed as the percentage change compared to baseline recording values) were analyzed using the non-parametric Wilcoxon signed-rank test when compared within one group, or the Mann-Whitney *U*-test when data were compared between groups. *P* < 0.05 was considered statistically significant. All statistical analyses were performed using Graph Pad Prism software (Graph Pad Software, Inc. San Diego, CA, USA).

## Results

### NMDA-induced LTD in distinct dendritic branches of CA1 neurons

Initially we examined the effect of NMDA application on LTD in distinct dendritic branches of hippocampal CA1 neurons: distal apical, proximal apical and basal, using field recordings. A schematic representation of transverse hippocampal CA1 lamina with stimulation electrode locations in proximal, distal and basal dendritic regions and the location of fEPSP recording electrodes in the corresponding regions is shown in Figure 1A. First we tested the effect of NMDA-induced LTD on synapses in distal apical dendrites of CA1 pyramidal neurons. Induction of LTD by bath application of 30 μM NMDA for 5 min resulted in a persistent form of LTD as reported previously (Lee et al., 1998; Kamal et al., 1999; Li et al., 2004). The LTD was maintained for at least 90 min compared with baseline values before NMDA application (Figure 1B: fEPSP value before NMDA application = 94.4 ± 1.3% of baseline, 90 min after = 56.8 ± 10.2% of baseline, *n* = 6, Wilcoxon signed-rank test, *p* = 0.031).

Next we again induced LTD by NMDA application, but recorded from proximal apical dendrites. The application of NMDA caused an initial depression in proximal apical dendrites followed by a return to baseline (Figure 1C: fEPSP before NMDA application = 96.6 ± 6.7% of baseline, 90 min after = 92.7 ± 7.7% of baseline, *n* = 6, Wilcoxon signed-rank test, *p* = 0.312). This suggests that the expression of LTD is distinct in distal vs. proximal dendrites of CA1 neurons, and this difference is due to cell-intrinsic (post-synaptic) properties of distal and proximal dendrites.

Next we investigated NMDA-induced LTD in basal dendrites (stratum oriens) of hippocampal CA1 neurons. Bath application of NMDA resulted in a persistent LTD in basal dendrites that lasted for at least 90 min (Figure 1D: fEPSP before NMDA application = 97.0 ± 3.7% of baseline, 90 min after = 74.1 ± 6.5% of baseline, *n* = 7, Wilcoxon signed-rank test, *p* = 0.015). However, the magnitude of basal dendritic LTD was less than that of distal apical dendritic LTD.

Thus, NMDA-induced LTD was not maintained in proximal apical dendrites, but was maintained in both distal apical dendrites and basal dendrites, with less depression in basal dendrites that in distal dendrites 30 min after induction (Figure 1E).

### Synaptically induced LTD in distinct dendritic branches of CA1 neurons

It is possible that NMDA bath application activates extra-synaptic NMDA receptors, and thus may not be a physiologically relevant method of LTD induction. We therefore compared synaptically (electrically)-induced LTD to NMDA-induced LTD in proximal and distal apical dendrites, and in basal dendrites of pyramidal neurons. First we induced electrical LTD in distal apical dendrites using a LFS protocol as described previously (Parvez et al., 2010). The LTD was maintained for at least 90 min compared with baseline values before LFS (Figure 2B, gray circles, fEPSP value before LFS = 98.78 ± 1.6% of baseline, 90 min after = 68.2 ± 3.6% of baseline, *n* = 6, Wilcoxon signed-rank test, *p* = 0.031), similar to NMDA-induced LTD in this region.

Next we again induced LTD by LFS, but recorded from proximal apical dendrites. LFS caused an initial depression in proximal apical dendrites followed by a return to baseline (Figure 2A, gray circles, fEPSP before LFS = 101.8 ± 2.7% of baseline, 90 min after = 99 ± 9% of baseline, *n* = 5, Wilcoxon signed-rank test, *p* = 0.812), again similar to NMDA-induced LTD in proximal apical dendrites.

Finally, we investigated LFS-induced LTD in basal dendrites (stratum oriens) of hippocampal CA1 neurons. LFS resulted in a persistent LTD in basal dendrites that lasted for at least 90 min (Figure 2C, gray circles, fEPSP before LFS = 99.05 ± 3.3% of baseline, 90 min after = 73.43 ± 7.9% of baseline, *n* = 4, Wilcoxon signed-rank test, *p* = 0.045). Similar to NMDA-induced LTD, the magnitude of proximal dendritic LFS-induced LTD was less than that of distal apical dendritic LFS-induced LTD.

Although NMDA induced a greater initial depression than LFS, both LFS and NMDA-induced LTD showed a similar magnitude in each lamina (Figure 2A: distal dendrites, *p* = 0.484 after 90 min, Figure 2B: proximal dendrites, *p* = 0.662 after 90 min, Figure 2C: basal dendrites, *p* = 0.171 after 90 min). These results indicate that the expression of LTD either by chemical (NMDA application) or electrical induction follows similar trends in the different lamina, i.e., LTD was not maintained in proximal apical dendrites, but was maintained in both distal apical dendrites and basal dendrites, with less depression in basal dendrites than in distal dendrites 30 min after induction (Figure 2C, gray circles). These results are consistent with previous reports that electrically induced LTD was maintained in distal dendrites of CA1 pyramidal but not in proximal dendrites (Parvez et al., 2010).

### NMDA induced LTD occludes further electrical LTD induction

Previous studies have shown that chemically induced LTD is saturable and occludes synaptically induced LTD at the same synapses (Lee et al., 1998). To verify this with our stimulation protocols, we induced NMDA LTD (Figure 3A, fEPSP value before NMDA LTD = 99.8 ± 2.2% of baseline, 60 min after = 64.9 ± 9.1% of baseline, *n* = 5, Wilcoxon signed-rank test, *p* = 0.01) followed by LFS 90 min after induction of chemical LTP. LFS did not induce further LTD; LFS-induced LTD was not maintained and returned to the level initially induced by NMDA within 60 min (Figure 3A, fEPSP value before LFS LTD = 64.9 ± 9.1% of baseline, 60 min after = 59.1 ± 10.7% of baseline, *n* = 5, Wilcoxon signed-rank test, *p* = 0.25). These data show that NMDA-induced LTD occludes LFS-induced LTD, and further suggests that NMDA and LFS-induced LTD share a common saturable expression mechanism.

### Inhibition of GABAergic activity blocks NMDA-induced LTD

Differential LTD expression in distinct dendritic lamina of hippocampal CA1 pyramidal neurons could be due to differences in intrinsic cell excitability or to differential GABAergic innervation and activity of interneurons in different layers, as previously hypothesized for apical dendritic LTD (Parvez et al., 2010). To address the latter possibility, we tested the effects of blocking GABAergic inhibition with picrotoxin (PTX) in distal apical and basal dendrites, the two regions in which LTD is maintained. Bath application of 50 μM PTX from 25 min before application of 30 uM NMDA and throughout the experiment, reduced the magnitude of initial LTD induction in distal apical dendrites, and this LTD decayed to baseline approximately 60 min after induction (Figures 3B,C, gray circles, fEPSP values before NMDA induced LTD = 94.1 ± 3.7% of baseline, 90 min after = 87.0 ± 7.9% of baseline, *n* = 4, Wilcoxon signed-rank test, *p* = 0.62).

Next we investigated the effects of PTX on NMDA-induced LTD in basal dendrites (stratum oriens). Bath application of 50 μM PTX also reduced the initial magnitude of LTD in this region, but the LTD decayed more quickly than in distal apical dendrites, and reached baseline within 20 min of induction (Figures 3D,E, fEPSP before NMDA application = 97.7 ± 4.1% of baseline, 90 min after = 95.1 ± 5.8 of baseline, *n* = 4, Wilcoxon signed-rank test, *p* = 0.62). Because LTD decayed in proximal dendrites in control conditions, and PTX caused decay of LTD in both distal apical and basal dendrites, we did not test the effects of PTX in proximal dendrites, where we would expect no effect (or an overshoot of baseline). These results indicate that GABAergic innervation is important for the maintenance of LTD in both distal apical and basal dendrites, but the time course of LTD decay in the absence of GABAergic input is different in each region.

### NMDA-induced LTD of the population spike response of CA1 neurons

Because long-term modification of synapses often reflects a change in the firing properties of the cell (Bernard and Wheal, 1995), we also examined the effect of NMDA-induced LTD on the population spike of pyramidal neurons, which represents the response in cell bodies. A schematic representation of transverse hippocampal CA1 lamina with test pulse stimulation electrode locations in proximal and distal dendritic regions in stratum radiatum, and in basal dendrites of stratum oriens of the CA1 region, and the location of the electrode for recording the population spike from the CA1 pyramidal neuron cell body layer is shown in Figure 4A. First we tested the effect of NMDA-induced LTD on the population spike response, when inputs to the distal apical dendrites in the stratum radiatum were given a test pulse. Bath application of 30 μM NMDA for 5 min resulted in LTD of the PSA. PSA LTD was maintained for at least 90 min compared with baseline values before NMDA application (Figure 4B: PSA value before NMDA application = 92.2 ± 2.4% of baseline, 90 min after = 55.9 ± 5.3% of baseline, *n* = 6, Wilcoxon signed-rank test, *p* = 0.031). When test pulses were given to inputs to the proximal dendrites in the stratum radiatum following NMDA-induced LTD, we observed an initial depression of the PSA followed by a return of potentials to baseline (Figure 4C: PSA before NMDA application = 107.5 ± 7.8% of baseline, 90 min after = 101.9 ± 10.2% of baseline, *n* = 6, Wilcoxon signed-rank test, *p* = 0.437), similar to the fEPSP response recorded from proximal dendrites (Figure 1C).

Finally we investigated the cell body response to NMDA-induced LTD when the test pulse was given to inputs to the basal dendrites of the stratum oriens of CA1 neurons. LTD induced by 30 μM NMDA application for 5 min showed an initial depression of the PSA, which returned to baseline within 60 min (Figure 4D: PSA before NMDA application = 107.8 ± 6.3% of baseline, 90 min after = 109.9 ± 15.6% of baseline, *n* = 6, Wilcoxon signed-rank test, *p* = 1). Thus the basal dendritic LTD recorded from basal dendrites was not maintained in the cell body.

In summary, the PSA response to distal apical dendrite stimulation after NMDA-induced LTD was maintained in the cell body, while the PSA response to proximal apical dendrite stimulation was not (Figure 4E), similar to the dendritic LTD recorded in these regions by fEPSP. The PSA response to stimulation of basal dendrites of the stratum oriens, on the other hand, was not maintained following NMDA-induced LTD, in contrast to the fEPSP response recorded from this region.

## Discussion

In this study we investigated whether distinct branches of hippocampal CA1 pyramidal neurons have cell-intrinsic post-synaptic differences in LTD expression. To date only a couple of studies have compared LTD expression in distinct dendritic regions—in one LTD was maintained in distal but not proximal apical dendrites (Parvez et al., 2010), and in another, no difference was found between apical and basal dendrite LTD, although proximal and distal dendrites were not distinguished in this study (Pavlowsky and Alarcon, 2012). To directly compare LTD expression in each dendritic compartment in CA1 hippocampal pyramidal neurons, and to bypass possible effects of differences in presynaptic inputs or stimulation to each compartment, we induced LTD globally by bath application of 30 μM NMDA for 5 min, which has previously been shown to result in LTD identical to that induced by LFS (Oliet et al., 1997; Kameyama et al., 1998; Lee et al., 1998; Kamal et al., 1999; Li et al., 2004).

We found that NMDA induced LTD of both dendritic and somatic responses at synapses onto distal apical dendrites of CA1 pyramidal neurons in stratum radiatum (Figures 1B, 4B), similar to previous reports using LFS-induced LTD (Dudek and Bear, 1992; Mulkey and Malenka, 1992; Bernard and Wheal, 1995).

In contrast, we found that in proximal dendrites NMDA-induced LTD resulted in an initial depression of both fEPSP and PSA, but these responses returned to baseline values within 30 min and LTD was not maintained (Figures 1C, 4C). This is similar to previous studies in which fEPSPs decayed in this region following LFS-induced LTD (Paulsen et al., 1993; Parvez et al., 2010). Our results suggest that the inability to maintain LTD in this region is a post-synaptic cell-intrinsic effect and is not caused by specific stimulation of inputs to proximal dendrites.

LTD induced by low frequency synaptic stimulation showed similar trends compared to NMDA-induced LTD, with no significant difference between NMDA and LFS induced LTD in each dendritic compartment tested (Figure 2). This suggests that NMDA-induced LTD recapitulates synaptically induced LTD and is physiologically relevant. To further verify this, we tested the effects of NMDA-induced LTD followed by LFS-induced LTD, and found that NMDA-induced LTD occluded LFS-induced LTD in our experiments, similar to previous reports (Oliet et al., 1997; Kameyama et al., 1998; Lee et al., 1998; Kamal et al., 1999; Li et al., 2004). The occlusion of LFS-induced LTD 90 min after NMDA-induced LTD (Figure 3A) further confirms that both forms of LTD use a common expression mechanism (Lee et al., 1998).

Why is LTD in proximal dendrites not maintained after induction? It has been hypothesized that L-type voltage-dependent calcium channel activation or locally generated, spatially restricted dendritic spikes in proximal dendrites could contribute to postsynaptic depolarization and calcium entry, which shifts the balance to LTP rather than LTD in this region (Golding et al., 2002; Parvez et al., 2010). In addition, proximal dendrites have more GABAergic innervation than distal apical branches (Megías et al., 2001), which may result in increased inhibition that blocks post-synaptic signaling cascades necessary for LTD. An increase in inhibition is likely in our experiments, since NMDAR activation itself has been reported to enhance inhibitory GABAergic transmission onto hippocampal pyramidal neurons (Xue et al., 2011). We tested the effects of GABAergic inhibition and found that NMDA-induced LTD decayed in both apical distal dendrites and basal dendrites in the presence of PTX to block GABAergic inputs, consistent with previous reports that LFS-induced LTD in stratum radiatum is blocked by PTX (Izumi et al., 2013). However, the time course of LTD decay was distinct in apical distal dendrites compared to basal dendrites (Figures 3B–E, gray filled circles). This is consistent with the notion that LTD is more easily induced in distal apical dendrites than in basal dendrites, perhaps due to differential inhibition. It takes longer for LTD to decay in distal apical than in basal dendrites when GABAergic inputs are blocked, suggesting that inhibition is more important for maintaining LTD in basal dendrites than in apical distal dendrites. PTX would be expected to have no effect on LTD in proximal apical dendrites, since LTD decayed in this region in control experiments.

The mechanisms of induction and maintenance of LTP are different in apical and basal dendrites of CA1 pyramidal neurons (Arai et al., 1994; Kramár and Lynch, 2003; Sajikumar et al., 2007; Navakkode et al., 2012; Fan, 2013). But LTD expression in apical vs. basal dendrites is not well studied. In basal dendrites, we found that NMDA-induced LTD was maintained for at least 90 min. As mentioned above, in proximal and distal apical dendrites the fEPSP and PSA responses were similar, exhibiting decay or maintenance respectively. But in basal dendrites, fEPSP LTD was maintained and PSA LTD was not. This is somewhat surprising given reports that fEPSP responses correlate with PSA (Daoudal et al., 2002), and that activity-dependent changes in neuronal excitability occur during LTP and LTD induction (Daoudal et al., 2002; Brager and Johnston, 2007). Since NMDA was bath applied, PSA evoked by stimulation of synapses in different dendritic subfields might therefore be expected to exhibit some depression. The fact that the PSA response to basal dendrite stimulation only exhibited a moderate degree of depression, which was not maintained, suggests differential expression of plasticity-induced excitability, likely via differences in ion channel type or distribution, in basal dendrites compared to apical dendrites. However, these experiments have the caveat that PSA recordings may report differences in neuronal excitability, but do not measure membrane potential directly. In any case, our results show that basal dendritic LTD is spatially confined as a result of cell-autonomous post-synaptic mechanisms. This spatial segregation is supported by previous reports that the firing pattern of the postsynaptic neuron can be changed without any change in the fEPSP slope (Bliss and Lomo, 1973; Abraham et al., 1985; Taube and Schwartzkroin, 1988).

In summary, the bulk of previous data suggests that LTP is more easily induced in proximal apical dendrites than in distal apical or basal dendrites, while we found that the opposite is true for LTD, which is more easily induced in distal apical dendrites and basal dendrites than in proximal apical dendrites. Our data further show that these differences in LTD expression are due to cell-intrinsic post-synaptic properties of CA1 pyramidal neuron dendrite sub-compartments.

## Conflict of interest statement

The authors declare that the research was conducted in the absence of any commercial or financial relationships that could be construed as a potential conflict of interest.
